# The Ascending Spirit

**Published:** 1891-06

**Authors:** 


### THE ASCENDING SPIRIT.

There have been accounts by *Seers* of the severance of the spirit from its earthly tenement at the period of death, and the following account by a French scientist, aided by a delicate apparatus, is wholly consistent with what these Seers claim to have observed.

The narrator was called to the bedside of a dying person. He had been expecting the summons, and remained beside the dying man until the approach of death became manifest.

"A sudden trembling, shaking the whole body, announced that the supreme moment had come. With one of my friends who was assisting me, we placed our heads under the dark covering of the apparatus and kept our eyes steadfastly fixed on the object glass. The particles of dust in the air were magnified many thousands of times, and for a moment their violent movement produced a cloud in front of the glass. Then a delicate column of violet vapor, condensed into a flocculent mass, was clearly seen above and around the body. Particles appeared to pursue one another as if obedient to some kind of central attraction. The cloud condensed more and more, and took the vaporous form of a man, then rapidly became purified until it was as colorless as the most perfect crystal.

At the time there was around us a feeling of terrible stillness—a calm that was almost agonizing. An indescribable sensation held us to the instrument, while our hearts seemed to cease pulsating. We kept our eyes fixed on the glass. Particle after particle grouped themselves together so as to produce the exact form of the man we knew so well. The form floated at about a foot above the body, to which it was distinctly united by a delicate cord. The face was undoubtedly the face of the man, but much finer and calmer. The eyes were closed, and the astral shape seemed to be asleep. By a double impulse, we, both of us, experienced the desire that the form should awake. At that very moment the bond which joined it to the body broke. A slight trembling passed over this beautiful, perfectly modelled form; a violet flame shone where the heart should be. It stood up and gave a sorrowing look at the abandoned body, extended the right hand with a gesture of adieu, then vanished, condensing into a small sphere which disappeared in the dawn of the everlasting to-morrow."

